# TET2 inhibits the proliferation and metastasis of lung adenocarcinoma cells via activation of the cGAS-STING signalling pathway

**DOI:** 10.1186/s12885-023-11343-x

**Published:** 2023-09-04

**Authors:** Gui Cheng, Jun Wu, Mei Ji, Wenwei Hu, Changping Wu, Jingting Jiang

**Affiliations:** Department of Tumor Biological Treatment, The Third Affiliated Hospital, Soochow University, 185 Juqian Street, Changzhou, 213003 P.R. China

**Keywords:** TET2, DNA hydroxymethylation, 5hmC, cGAS-STING signaling, Non-Small Cell Lung Cancer

## Abstract

**Background:**

Effective identification and development of new molecular methods for the diagnosis, treatment and prognosis of lung adenocarcinoma (LUAD) remains an urgent clinical need. DNA methylation patterns at cytosine bases in the genome are closely related to gene expression, and abnormal DNA methylation is frequently observed in various cancers. The ten-eleven translocation (TET) enzymes oxidize 5-methylcytosine (5mC) and promote locus-specific DNA methylation reversal. This study aimed to explore the role of the TET2 protein and its downstream effector, 5-hmC/5-mC DNA modification, in LUAD progression.

**Methods:**

The expression of TET2 was analysed by real-time PCR, Western blotting and immunohistochemistry. The 5-hmC DNA content was determined by a colorimetric kit. Activation of the cGAS-STING signalling pathway was evaluated by Western blotting. CCK-8, wound healing and Transwell assays were performed to evaluate the effect of TET2 on cell proliferation, migration and invasion abilities. A xenograft model was used to analyse the effect of TET2 on the tumorigenic ability of A549 cells.

**Results:**

TET2 overexpression decreased proliferation and metastasis of A549 and H1975 cells in vitro and in vivo. However, TET2 knockdown dramatically enhanced the proliferation, migration and invasion of A549 and H1975 cells. Mechanistically, activation of the cGAS-STING signalling pathway is critical for the TET2-mediated suppression of LUAD cell tumorigenesis and metastasis.

**Conclusion:**

In this study, we demonstrate a tumour suppressor role of TET2 in LUAD, providing new potential molecular therapeutic targets and clinical therapies for patients with non-small cell lung cancer.

**Supplementary Information:**

The online version contains supplementary material available at 10.1186/s12885-023-11343-x.

## Introduction

Lung cancer, including non-small cell lung cancer and small cell lung cancer, is the leading cause of cancer-related deaths worldwide [1, 2]. non-small cell lung cancer accounts for approximately 80% of all lung cancers, and the main histological subtypes are LUAD and lung squamous cell carcinoma [3]. Despite various treatment strategies, its prognosis remains very poor, with a 5-year survival rate of less than 15% [4]. Therefore, the effective identification and development of new molecular methods for LUAD diagnosis, treatment and prognosis remains an urgent clinical need.

DNA methylation patterns at cytosine bases in the genome are closely related to gene expression, and abnormal DNA methylation is frequently observed in diseases [5]. TET enzymes oxidize 5mC and promote locus-specific DNA methylation reversal [6]. In mammalian cells, all members of the TET family (TET1, TET2, and TET3) catalyse the continuous oxidation of 5mC to produce 5hmC, 5-formylcytosine (5fC), and 5-carboxylic cytosine (5caC) [7]. Under physiological conditions, TET enzymes play important roles in cell fate determination, cell differentiation and development [8]. From a pathological perspective, inactivating mutations in TET genes, mainly TET2, frequently occur in various cancers, including haematopoietic malignancies of bone marrow and the lymphatic system and some solid tumours (breast and colorectal cancer) [9–13]. The TET2-interacting proteins IDAX/CXXC4 and RINF/CXXC5 are overexpressed in a number of solid tumours, including breast cancer and colorectal cancer, and have been reported to negatively affect TET2 stability and function [14]. In addition, several microRNAs overexpressed in cancer have been reported to directly target TET proteins [15, 16]. In particular, miR-22 has a negative regulatory effect on all three members of the TET family [17]. Its expression is also associated with poor clinical prognosis in breast cancer patients [18]. However, the association between TET2 and LUAD largely remains unclear.

The cyclic GMP-AMP synthase (cGAS)-stimulator of interferon genes (STING) pathway has emerged as a critical innate immune pathway that, following engagement by DNA, promotes distinct immune responses [19, 20]. In recent years, accumulated evidence has suggested that dysregulation of the cGAS-STING pathway is involved in cancer pathogenesis, including LUAD [21, 22]. Numerous studies have demonstrated that aberrant activation of the cGAS-STING pathway promotes lung cancer cell proliferation, survival, and invasion, thus contributing to tumour growth and metastasis [21, 23, 24]. Additionally, impaired activation of this pathway leads to reduced immune-mediated tumour clearance, highlighting its critical role in modulating anti-tumour immunity. Given the importance of the cGAS-STING pathway in cancer biology, novel strategies targeting this pathway have shown promising results and are currently under investigation for potential clinical utility in cancer immunotherapy [25, 26]. The latest research has indicated that combining cGAS-STING pathway-targeted agents with other immunotherapeutic approaches, such as immune checkpoint inhibitors, may result in enhanced anti-tumour efficacy, further highlighting the potential of this pathway in personalized cancer therapies [23].

In this study, we demonstrate that TET2-mediated DNA hydroxymethylation inhibits the proliferation and metastasis of lung cancer cells in vitro and in vivo. Moreover, we found that cGAS-STING signalling activation is critical for TET2-mediated inhibition of cell proliferation and metastasis in LUAD.

## Materials and methods

### Human LUAD tissue specimens

A total of 60 LUAD tissues and matched adjacent normal tissues collected at the Third Affiliated Hospital of Soochow University between 2015 and 2018 were obtained. The clinicopathological characteristics of the 60 LUAD patients were presented in Table 1. Tissue samples were obtained during lung resection surgery and stored in liquid nitrogen at -80 °C. This study was approved by the Ethics Committee of the Third Affiliated Hospital of Soochow University (Changzhou, China; No. 2,023,054). All patients gave informed consent.

**Table 1:** The clinicopathological characteristics of the 60 LUAD patients

Characteristic		Whole cohort
Gender		
	Male	35
	Female	25
Age [years; median (range)]		62 (37.0–75.0)
Histological grade		
	G1	8
	G2	34
	G3	18
Tumor stage		
	T1	31
	T2	22
	T3	4
	T4	3
Nodal stage		
	N0	37
	N1	13
	N2	8
		2
Metastasis stage		
	M0	58
	M1	2
Clinical stage (AJCC 8th )		
	I	27
	II	18
	III	15

### Cell lines and cell culture

All cells in this study were obtained from the Chinese Academy of Sciences, Shanghai Institutes for Biological Sciences. HEK293 cells and A549 and H1975 LUAD cells were cultured in DMEM supplemented with 10% foetal bovine serum (FBS).

### Expression plasmids and lentivirus

Full-length human TET2 cDNA was inserted into the pLVX-Ires-Puro vector (Clontech) to construct the TET2 overexpression plasmid. The siRNA targeting TET2 was purchased from Genelily BioTech Co., Ltd (Shanghai, China). To establish individual stable call lines, lentiviruses were used. After transfection of lentiviral vectors into HEK293 cells for 72 h, lentiviral particles in the cell culture supernatant were collected. A549 and H1975 cells were then infected with these lentiviral particles. The stably transfected cells were identified by screening with puromycin (2 µg/mL) for 1 week beginning 48 h after lentiviral infection.

### Cell proliferation assay

The cell proliferation assay was performed using a Cell Counting Kit-8 (CCK-8) (Dojindo, Japan) in accordance with the manufacturer’s instructions. Cells (1 × 10^4^ cells/mL) were seeded in 96-well plates (100 µL/well) and cultured in a 5% CO_2_ incubator at 37 °C for 24, 48, and 72 h. Ten microlitres of CCK-8 solution was added to each well and incubated for 2 h at 37 °C. The absorbance of each sample at 450 nm was measured with a spectrophotometer.

### Transwell assay

The invasiveness of cells was evaluated by the Martrigel (BD Bioscience)-coated Transwell (Corning Inc.). Cells (1 × 10^5^) were seeded in the upper chamber in serum-free DMEM, and DMEM containing 20% FBS was added to the lower chamber. After incubation for 48 h, the cells on the upper surface of the membrane in the upper chamber were gently removed with a cotton swab. Then, the cells on the bottom surface of the membrane were fixed with 4% paraformaldehyde (PFA) for 15 min and stained with 0.1% crystal violet for 10 min. After washing with PBS 3 times, the invaded cells on the filter membrane were imaged, and cells in 5 random images were counted under the microscope to determine the number of invaded cells.

### Wound healing assay

The cell migration ability was measured by a wound healing assay. A total of 10^5^cells per well were seeded in 6-well plates. When the cell confluence was approximately 90%, an artificial wound was made in the cell layer with a 200 µL pipette tip. Twenty-four hours later, wound healing was observed, and imaging was performed under a microscope. The distance travelled by the cells is expressed as the percentage of cell coverage across the initial wound demarcation.

### RNA isolation and real-time PCR

Total RNA was extracted using TRIzol according to the manufacturer’s instructions. First-strand cDNA was synthesized using upper standard II (Invitrogen), using 1 µg total RNA for each cDNA synthesis reaction. Real-time PCR was performed using SYBR Green Universal Master Mix (Roche) and a primer mixture. GAPDH was used as the internal reference for mRNA expression. The primers used for real-time qPCR were presented in Table 2.

**Table 2:** The sequence of the primers used in real-time PCR.

Genes	Forwards Primer (5’-3’)	Reverse Primer (5’-3’)
TET2	GCCACTACCACACCACCACC	GCATCGGAGAAGGGCTGCAT
cGAS	CAGAAAAAGAGCGCCCCGGA	TCTTGGCTTCGTGGAGCAGC
STING	GCCACCCCCTTGCAGACTTT	CCTGGTAGGCAATGAGGCGG
TBK1	CAACCTGGAAGCGGCAGAGT	GCGTCTGCCAGTGATCCACC
GAPDH	CACCATCTTCCAGGAGCGAG	AAATGAGCCCCAGCCTTCTC

### Western blot analysis

Total protein was extracted and separated on SDS-PAGE gels. Antibodies specific for TET2 (1:2000), cGAS (1:1000), STING (1:1000), and TBK1 (1:1500) (all from ABclonal, Shanghai, China) were used. An anti-GAPDH antibody (Kancheng Biotechnology, 1:5000) was used as a loading control.

### Xenograft models

BALB/c nude mice (5 weeks of age) were purchased from SLAC Animal Center in Shanghai, China, for xenograft model establishment. Animal experiments were approved by the Animal Care Use Committee of the Third Affiliated Hospital of Soochow University. A total of 10^7^ A549 cells per mouse were injected subcutaneously and into the caudal vein of nude mice to evaluate the proliferation of lung cancer cells. The tumour volume was calculated every 5 days and was estimated from the length and width measurements as follows: Tumour volume = (length x width^2^)/2. After approximately 30 days, the nude mice were killed, and the tumours were then removed, photographed and weighed. In addition, A549 cells were injected into the tail vein of nude mice to evaluate the metastatic ability of lung cancer cells. Approximately 2 weeks after cell injection, the mice were sacrificed, and the number of lung metastases was determined.

### Statistical analysis

All experiments were repeated three times using independent cell cultures. GraphPad Prism 8.0 was used for statistical analysis. The data in all figures are expressed as the means ± SEMs. Student’s t test and two-way ANOVA were used to confirm that the differences were statistically significant. Overall survival was analysed by the Kaplan‒Meier method with the log-rank test. For all statistical tests, a significance level of less than 0.05 (two-tailed) was accepted as statistically significant.

## Results

### The TET2 expression and TET2-mediated 5-hmC modification levels are decreased in LUAD tissues

**Fig. 1:**
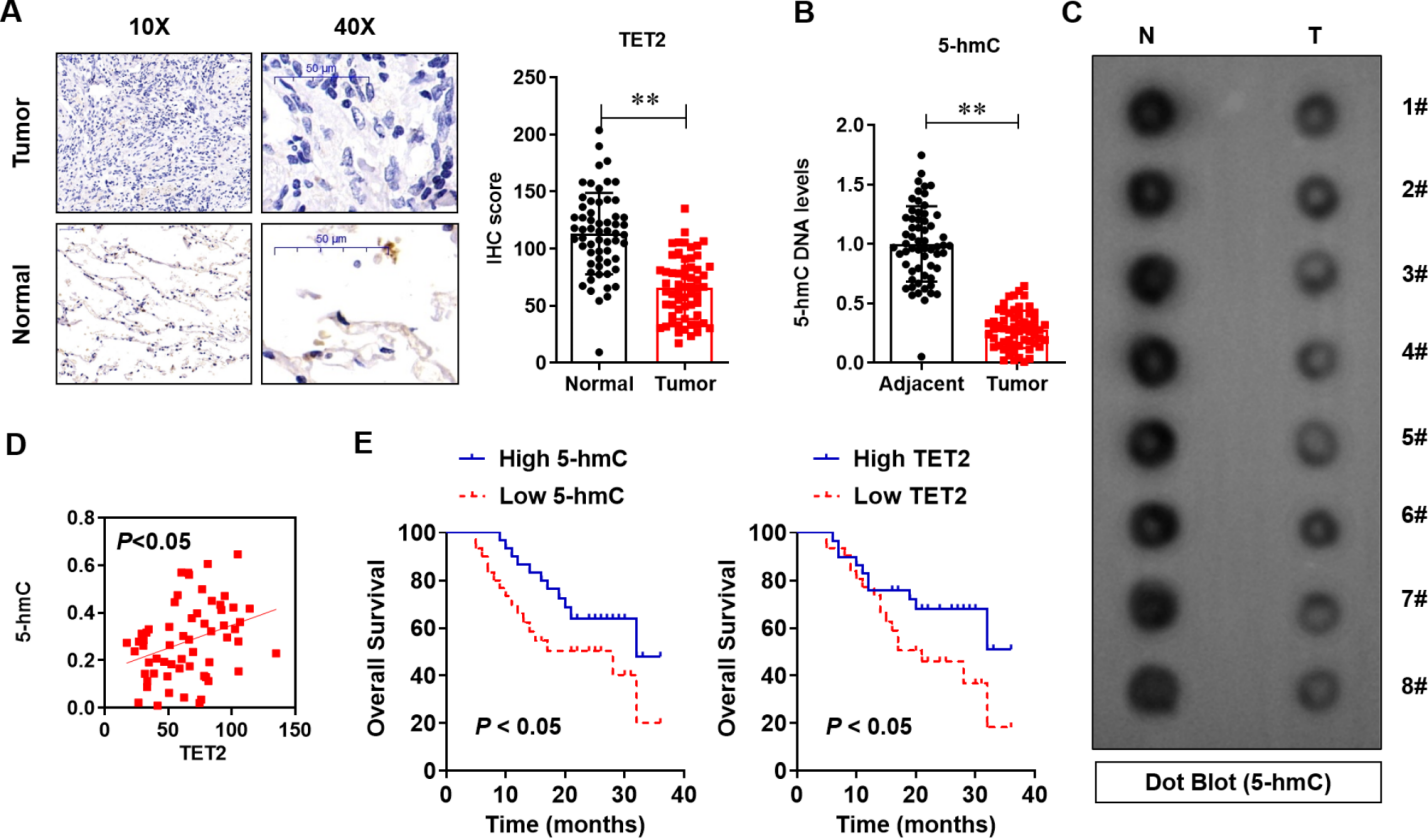
TET2 expression and TET2-mediated 5-hmC modification levels were significantly reduced in lung cancer tissues. **(A)** Immunohistochemical determination of TET2 protein expression levels in lung cancer and adjacent tissues; **(B)** Colorimetric assay to measure 5-hmC levels in lung cancer tissues and adjacent tissues; **(C)** The level of 5-hmC in lung cancer tissues and adjacent tissues was determined by dot blotting; **(D)** The correlation between the 5-hmC and TET2 level in lung cancer tissues; **(E)**The correlation between the 5-hmC and TET2 level with the prognosis of patients; **P < 0.01 versus indicated group

To understand the potential role of TET2 expression and TET2-mediated 5-hmC modification in LUAD, we first analysed the differences in TET2 expression and 5-hmC levels between adjacent normal tissues and tumours in 60 paired samples. Immunohistochemical staining and quantitative analysis of the data showed that TET2 protein levels were clearly lower in human lung cancer tissues than in normal adjacent lung tissues (*P* < 0.01, Fig. 1A). Consistent with this finding, the content of 5-hmC-modified DNA detected by the Global 5-hmC DNA Quantification Kit was lower in human lung cancer tissues than in normal adjacent lung tissues (*P* < 0.01, Fig. 1B), and this finding was further verified by dot blotting in 8 paired adjacent normal tissues and tumour tissues (Fig. 1C). Pearson correlation assay showed a positive correlation between TET2 and 5-hmC levels (Fig. 1D). Moreover, the prognostic correlation between the 5-hmC/TET2 levels and survival in lung cancer patients was analysed. Kaplan–Meier analysis indicated that lower 5-hmC levels were highly correlated with shorter overall survival times (*P* < 0.05, Fig. 1E). These results demonstrate that the levels of TET2 expression and TET2-mediated 5-hmC modification are decreased in lung cancer tissues.

### TET2 impairs the proliferation, migration and invasion of LUAD cells

**Fig. 2:**
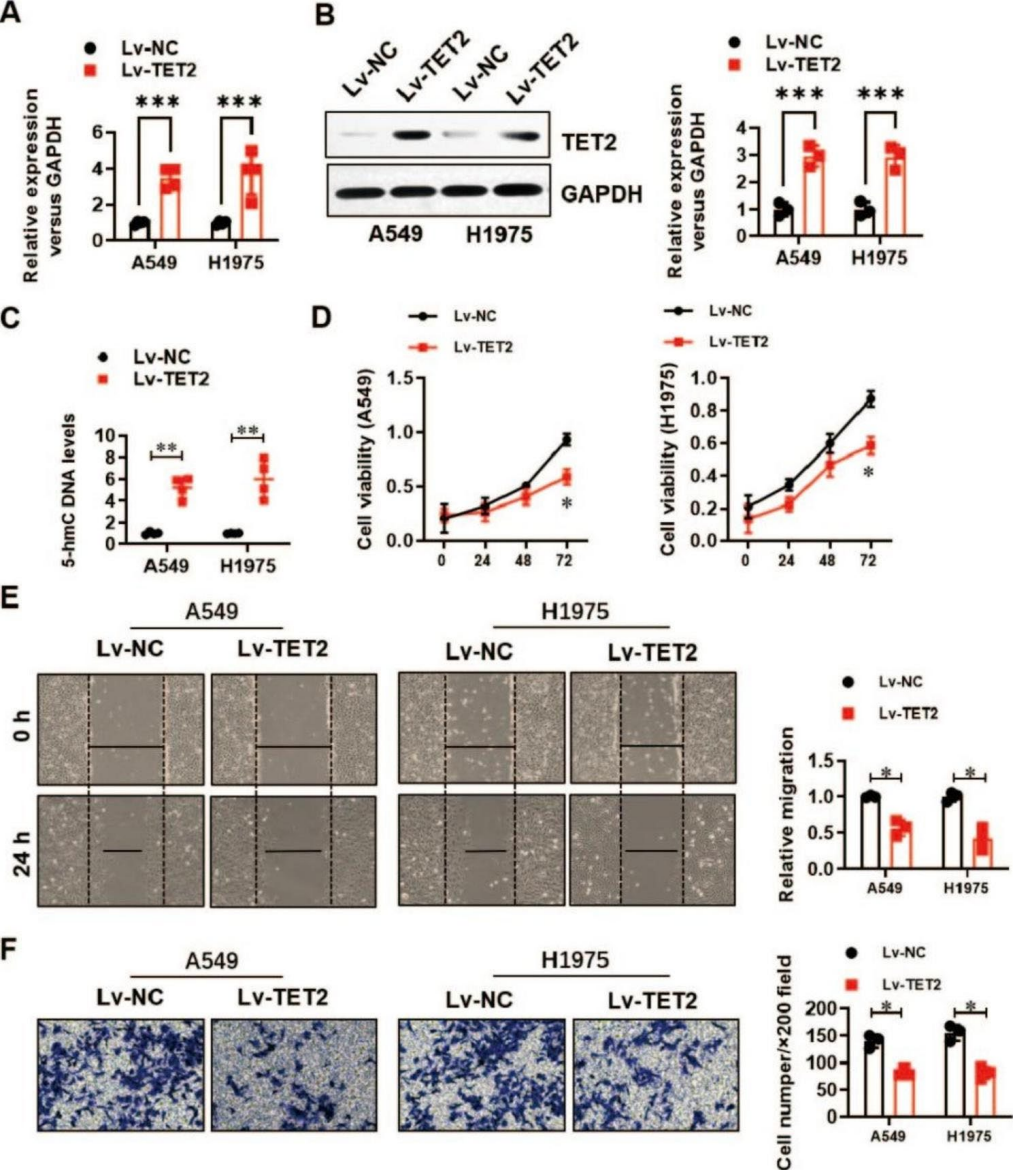
TET2 overexpression significantly inhibited the proliferation, migration and invasion of lung cancer cells. **(A)** Real-time PCR was used to verify the overexpression efficiency of TET2 in A549 and H1975 lung cancer cells; **(B)** Western blotting was used to verify the overexpression efficiency of TET2 in A549 and H1975 lung cancer cells; **(C)** Colorimetric verification of the effect of TET2 overexpression on 5-hmC levels in A549 and H1975 lung cancer cells; **(D)** The effect of TET2 overexpression on the proliferation of A549 and H1975 lung cancer cells was analysed by CCK-8 assay; **(E)** The effect of TET2 overexpression on the migration of A549 and H1975 lung cancer cells was analysed by a wound healing assay; **(F)** The effect of TET2 overexpression on the invasion ability of A549 and H1975 lung cancer cells was evaluated by a Transwell assay. *P < 0.05, **P < 0.01,***P < 0.001 versus indicated group

**Fig. 3:**
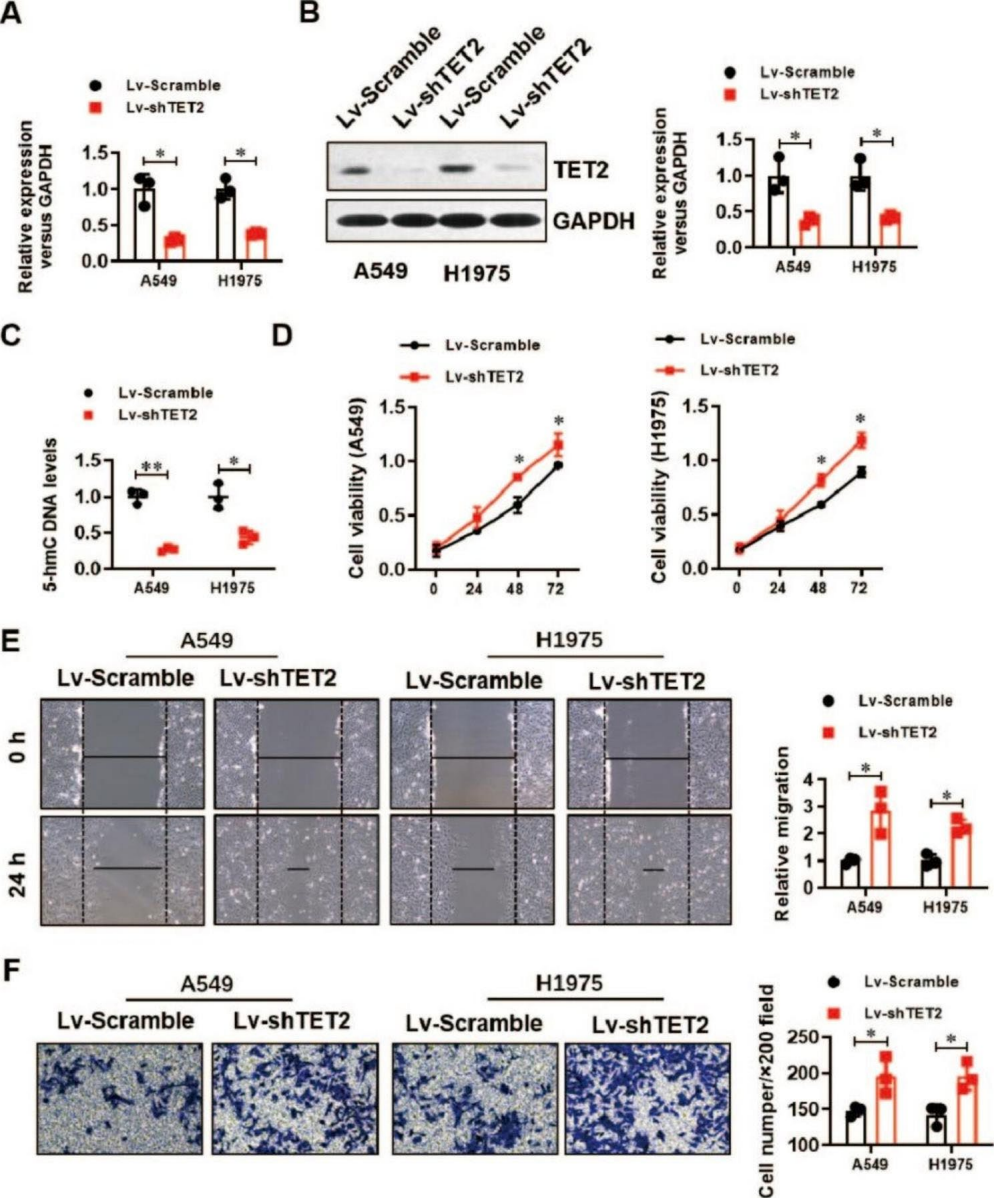
Downregulation of TET2 significantly enhanced the proliferation, migration and invasion of lung cancer cells. **(A)** Real-time PCR validation of the knockdown efficiency of TET2 in A549 and H1975 lung cancer cells; **(B)** Western blotting was used to verify the knockdown efficiency of TET2 in A549 and H1975 lung cancer cells; **(C)** Colorimetric verification of the effect of TET2 knockdown on 5-hmC levels in A549 and H1975 lung cancer cells; **(D)** The effect of TET2 knockdown on the proliferation of A549 and H1975 lung cancer cells was analysed by a CCK-8 assay; **(E)** The effect of TET2 knockdown on the migration of A549 and H1975 lung cancer cells was analysed by a wound healing assay; **(F)** The effect of TET2 knockdown on the invasion ability of A549 and H1975 lung cancer cells was evaluated by a Transwell assay. *P < 0.05, **P < 0.01 versus indicated group

To evaluate the role of TET2 in the biological behaviours of LUAD cells, we generated two LUAD cell lines (A549 and H1975) with stable TET2 overexpression. The overexpression efficiency was analysed by real-time PCR (Fig. 2A) and verified by Western blotting (Fig. 2B). The 5-hmC-modified DNA levels were significantly elevated by TET2 overexpression in the A549 and H1975 cell lines (Fig. 2C). TET2 overexpression dramatically inhibited the proliferation (Fig. 2D), migration (Fig. 2E) and invasion (Fig. 2F) of A549 and H1975 cells, as determined by CCK-8, wound healing and Transwell assays, respectively. In addition, we generated two TET2 knockdown cell lines (A549 and H1975). The knockdown efficiency was confirmed by real-time PCR (Fig. 3A) and Western blotting (Fig. 3B). Consistent with the above findings, the 5-hmC-modified DNA levels were significantly decreased by TET2 knockdown in A549 and H1975 cells (Fig. 3C). TET2 knockdown significantly promoted the proliferation (Fig. 3D), migration (Fig. 3E) and invasion (Fig. 3F) of A549 and H1975 cells. These results suggest a tumour suppressor role of TET2 in LUAD.

### TET2 significantly activates the cGAS-STING signalling pathway

**Fig. 4:**
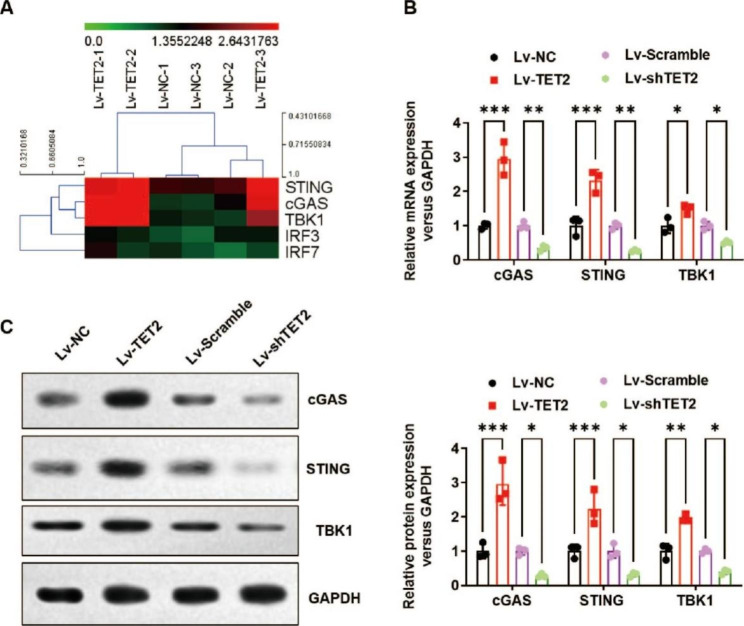
TET2 significantly activates the cGAS-STING signalling pathway. **(A)** RNA-seq analysis was performed, and the heatmap indicates the influence of TET2 overexpression on the expression levels of cGAS-STING signalling pathway-related genes (STING, cGAS, TBK1, IRF3 and IRF7) in A549 cells; **(B)** The effect of TET2 overexpression and knockdown on the mRNA expression levels of cGAS-STING signalling pathway-related genes (cGAS, STING and TBK1) in A549 cells was verified by real-time PCR; **(C)** Western blotting was used to verify the effect of TET2 overexpression and knockdown on the protein expression levels of cGAS-STING signalling pathway-related genes (cGAS, STING and TBK1) in A549 cells. *P < 0.05, **P < 0.01 versus indicated group

To further explore the regulatory mechanism of TET2 in LUAD, we analysed dysregulated transcription in TET2-overexpressing A549 cell lines and normal vector control cell lines using RNA sequencing. We found dramatically elevated expression of cGAS-STING signalling molecules, including c*G*AS, STING and TBK1, as shown in the heatmap (Fig. 4A). The elevated expression of cGAS, STING and TBK1 was verified by real-time PCR (Fig. 4B) and Western blotting (Fig. 4C). Consistently, TET2 knockdown significantly decreased the expression level of cGAS, STING and TBK1 in A549 cells (Fig. 4B and C).

### TET2 impairs the proliferation, migration and invasion of LUAD cells via activation of the cGAS-STING signalling pathway in vitro

**Fig. 5:**
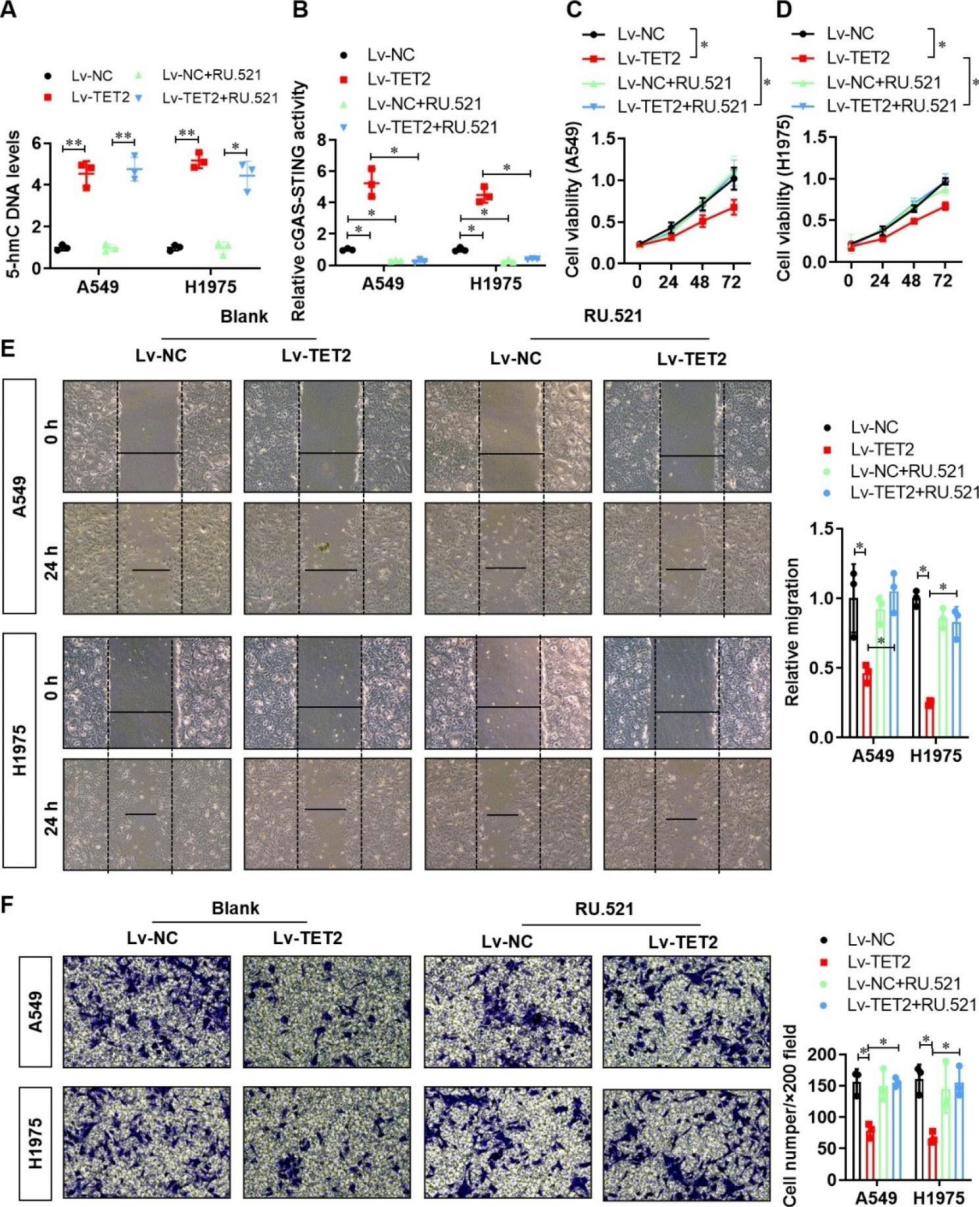
Inhibition of the cGAS-STING signalling pathway significantly reversed the inhibitory effect of TET2 overexpression on the proliferation, migration and invasion of lung cancer cells. **(A)** Colorimetric verification of the effects of the cGAS-STING signalling pathway inhibitor RU.521 and TET2 overexpression on 5-hmC levels and **(B)** the activity of the cGAS-STING signalling pathway in A549 and H1975 lung cancer cells; **(C)** The effects of the cGAS-STING signalling pathway inhibitor RU.521 and TET2 overexpression on the proliferation of A549 and **(D)** H1975 lung cancer cells were evaluated by a CCK-8 assay. **(E)** The effects of the cGAS-STING signalling pathway inhibitor RU.521 and TET2 overexpression on the migration ability of A549 and H1975 lung cancer cells were evaluated by a wound healing assay; **(F)** A Transwell assay was performed to evaluate the effects of the cGAS-STING signalling pathway inhibitor RU.521 and TET2 overexpression on the invasion ability of A549 and H1975 lung cancer cells. *P < 0.05, **P < 0.01 versus indicated group

To identify the contribution of cGAS-STING signalling to the TET2-medicated inhibition of lung cancer cell proliferation, migration and invasion, we then treated the vector control and TET2-overexpressing A549 and H1975 cell lines with the cGAS-STING signalling inhibitor RU.521 (10 µM). The elevated levels of 5-hmC-modified DNA in the TET2-overexpressing A549 and H1975 cell lines were unchanged by RU.521 treatment (Fig. 5A), whereas the activity of cGAS-STING signalling was clearly suppressed by RU.521 treatment (Fig. 5B). On the other hand, RU.521 treatment showed no effect on proliferation (Fig. 5C and D), migration (Fig. 5E) or invasion (Fig. 5F) of the vector control A549 and H1975 cell lines. However, TET2-mediated inhibition of proliferation (Fig. 5C and D), migration (Fig. 5E) and invasion (Fig. 5F) was clearly reversed by RU.521 treatment. These results demonstrate that TET2 impairs the proliferation, migration and invasion of lung cancer cells via activation of the cGAS-STING signalling pathway in vitro.

### TET2 impairs the tumorigenesis and metastasis of lung cancer cells via activation of the cGAS-STING signalling pathway in vivo

**Fig. 6:**
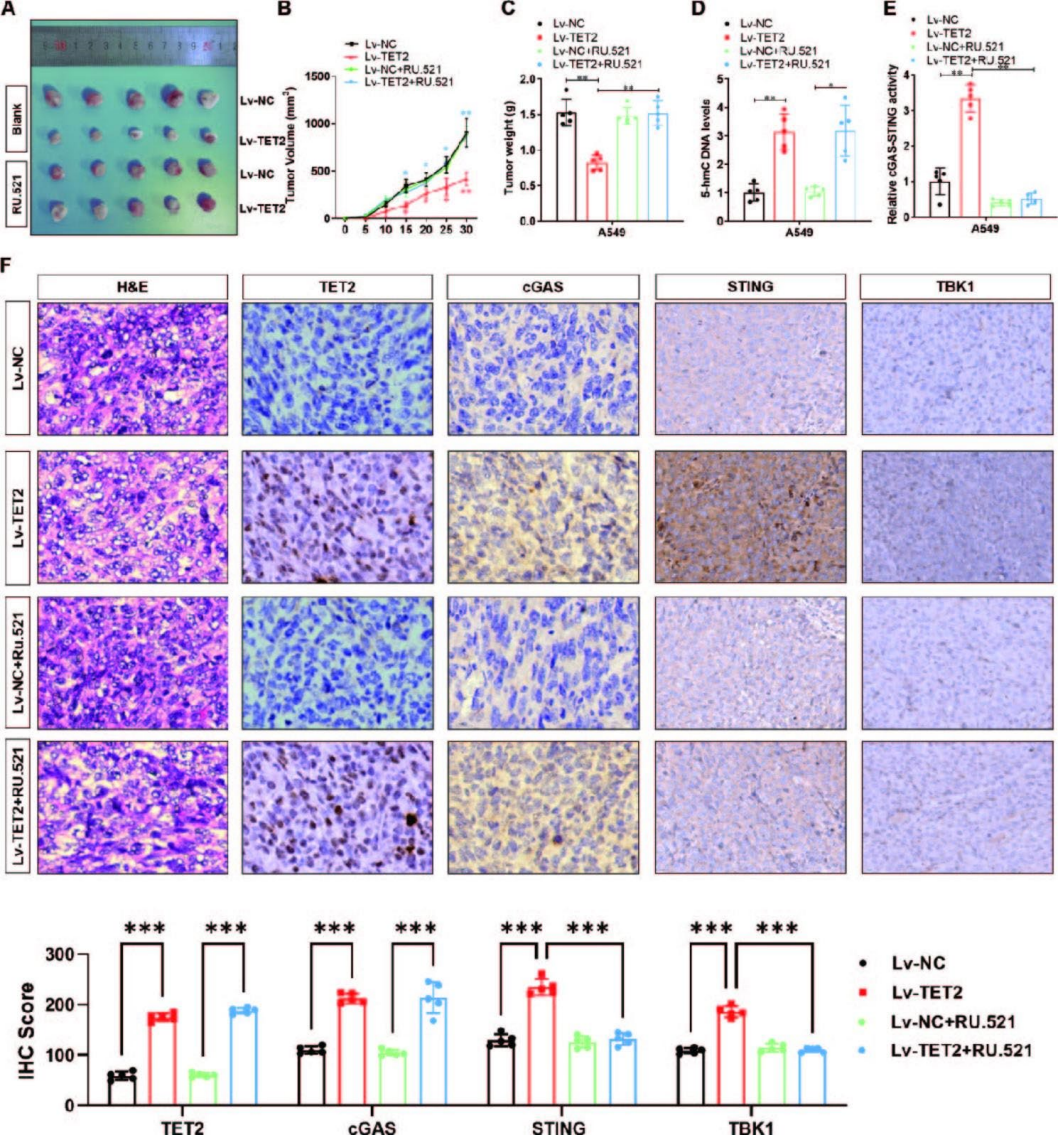
TET2 impairs the tumorigenesis of lung cancer cells via activation of the cGAS-STING signalling pathway in vivo. **(A)** Representation of lung cancer tumours generated by subcutaneous implantation of A549 cells in BALB/c NOD mice; **(B)** Tumour volume and growth were analysed; **(C)** Tumour weight was analysed; **(D)** Colorimetric quantification was used to verify the effect of TET2 overexpression on the 5-hmC level and **(E)** the activity of the cGAS-STING signalling pathway during tumour formation from A549 cells in nude mice; **(F)** Differences in the protein levels of TET2, cGAS, STING and TBK1 were verified by immunohistochemistry. *P < 0.05, **P < 0.01 versus indicated group

To further confirm the critical role of cGAS-STING signalling in TET2-mediated inhibition of lung cancer cell proliferation and metastasis in vivo, we generated a subcutaneous xenograft model in BALB/c NOD mice (n = 5 group) using vector control and TET2 overexpression cells. The mice were intraperitoneally injected with a cGAS-STING inhibitor (RU.521, 5 mg/kg) or normal saline every 5 days after assessment of the tumour volume. Clearly, the tumours derived from TET2-overexpressing A549 cells were smaller than those derived from the vector control A549 cells (Fig. 6A). Similar results were observed for the tumour volume (Fig. 6B) and tumour weight (Fig. 6C). The TET2-mediated increase in the 5-hmC-modified DNA level was unchanged by RU.521 treatment (Fig. 6D), whereas the activity of cGAS-STING signalling was dramatically suppressed by RU.521 treatment (Fig. 6E). Consistent with this finding, RU.521 treatment did not alter tumour growth in the vector control group, whereas it significantly increased tumour volume (Fig. 6B) and tumour weight (Fig. 6C) in the TET2 overexpression group. IHC staining further showed increased protein levels of TET2, cGAS, STING and TBK1 in TET2-overexpressing tumours, in which the protein levels of STING and TBK1 were clearly decreased by RU.521 treatment (Fig. 6F).

**Fig. 7:**
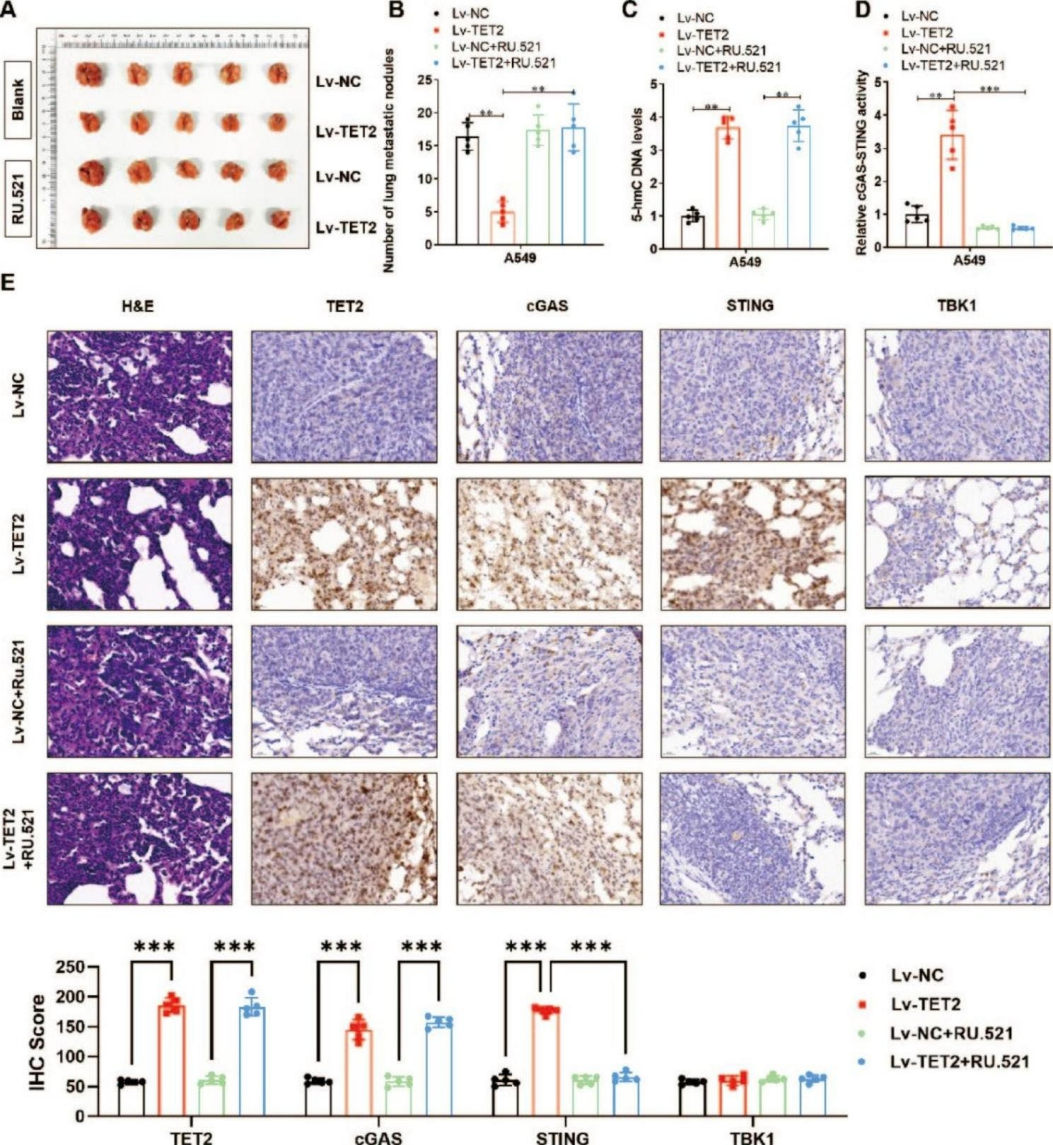
TET2 impairs the metastasis of lung cancer cells via activation of the cGAS-STING signalling pathway in vivo. **(A)** Lung cancer metastatic lesions were generated by tail vein injection of A549 cells in BALB/c NOD mice; **(B)** The number of lung metastatic lesions; **(C)** Colorimetric quantification was used to verify the effect of TET2 overexpression on the 5-hmC level and **(D)** the activity of the cGAS-STING signalling pathway in lung metastatic lesions formed from A549 cells in nude mice; **(E)** Differences in the protein levels of TET2, cGAS, STING and TBK1 were verified by immunohistochemistry. *P < 0.05, **P < 0.01,***P < 0.001 versus indicated group

We also used these cells to generate a mouse xenograft model of tumour metastasis via tail vein injection of A549 cells into BALB/c NOD mice (n = 5 group). The mice were also intraperitoneally injected with a cGAS-STING inhibitor (RU.521, 5 mg/kg) or normal saline every 5 days. Approximately 2 weeks after cell injection, the mice were sacrificed, and the lung metastatic lesions (Fig. 7A) were counted. The number of lung metastatic lesions in the TET2 overexpression group was significantly less than that in the vector control group, and, similar to the results described above, lung metastasis was reversed by RU.521 treatment (Fig. 7B). Consistent with this finding, the TET2-mediated increase in the 5-hmC-modified DNA level in lung metastatic lesions was unchanged by RU.521 treatment (Fig. 7C), whereas the activity of cGAS-STING signalling was significantly suppressed by RU.521 treatment (Fig. 7D). Moreover, IHC staining showed increased protein levels of TET2, cGAS, STING and TBK1 in TET2-overexpressing tumours, in which the protein levels of STING and TBK1 were clearly decreased by RU.521 treatment (Fig. 7E). Collectively, these results demonstrate that TET2 inhibits the tumorigenesis and metastasis of LUAD cells via activation of the cGAS-STING signalling pathway *in vivo.*

## Discussion

Previous studies have shown that reduced expression of the TET protein and reduced levels of 5-hmC are common features of gastric cancer, prostate cancer, liver cancer, lung cancer, breast cancer, glioblastoma, melanoma and other cancers [27–32]. Due to the severity of this phenomenon, it is unlikely that the observed reduction in 5-hmC is solely the effect of deleterious TET mutations [33]. Interestingly, assessment of the growth rate and 5-hmC content in a wide range of tissues revealed a significant negative correlation between the proliferation rate and the global level of 5-hmC [34]. Thus, the decline in the 5-hmC level may be attributed at least in part to the high proliferation rate of cancer cells, which leads to a general inability to maintain tissue-specific 5-hmC levels [35]. These observations suggest that mutations of TET proteins, high cell proliferation rates and changes in the levels of regulatory factors may lead to epigenetic degradation of 5-hmC and 5-mC patterns [36]. However, the exact effect of changes in TET activity on the transformation, progression and maintenance of these kinds of tumours is largely unknown and remains an active research topic. In this study, we demonstrated a tumour suppressor role of TET2 in LUAD. TET2 overexpression in A549 and H1975 cells clearly decreased their proliferation and metastasis in vitro and in vivo. In contrast, TET2 knockdown dramatically enhanced the proliferation, migration and invasion of A549 and H1975 cells.

The physiological correlation of TET protein expression with development was studied in a knockout mouse model. In mutant mice with defects in both TET1 and TET2, it was noted that while some mice were able to survive and develop normally, most died during the perinatal period and showed a wide range of defects, including anencephaly, growth retardation, and haemorrhage [37]. The complexity of this phenotype, including its partial penetration and high variability, strongly suggests that the defects cannot be explained by disruption of a single process or pathway [37]. The TET2 protein and its downstream effector, 5-hmC/5-mC DNA modification, are present in immune cells, including T cells, B cells and macrophages, under both physiological and pathological conditions [38–42]. TET2 has also been shown to play a key role in tumour progression. For example, Bonvin et al. reported that epigenetic regulation of TET2-dependent downstream genes impaired the initiation and progression of melanoma [43]. Kunimoto et al. reported that TET2 deletion and Nras mutations jointly promoted myeloid transformation [44, 45]. However, the downstream regulatory mechanism of TET2 in cancer remains unclear. In this study, we found that TET2 overexpression clearly activated the cGAS-STING signalling pathway.

Cyclic GMP-AMP synthase (cGAS) interferon gene (STING) signalling induces the expression of type I interferons (IFNs) and other inflammatory cytokines to activate the antibacterial innate immune defence in response to detection of viral and bacterial DNA [46]. In addition to pathogen recognition, cGAS-STING signalling is also important for tumour immunity because it induces type I IFN expression and activates innate and adaptive immunity [47]. In most cancer types studied, the expression levels of these genes were inversely correlated with their methylation levels [48]. To evade this DNA detection pathway to survive, there are several mechanisms used by tumour cells to generate defective cGAS-STING signalling, including reductions in the protein levels of cGAS and STING, hypermethylation of cGAS and STING promoter regions, and defects in STING translocation to the Golgi body, its usual signalling site [49, 50]. In this study, we found that the mRNA and protein levels of cGAS, STING and TBK1 were elevated by TET2 overexpression in A549 and H1975 cell lines, consistent with the epigenetically regulated role of TET2 in gene expression. Moreover, we inhibited the activity of cGAS-STING signalling with the specific inhibitor RU.521 and found that the TET2-mediated impairment of the biological functions of LUAD was totally reversed. Therefore, these results demonstrate that activating the cGAS-STING signalling pathway contributes greatly to TET2-mediated suppression of the tumorigenesis and metastasis of lung cancer cells.

## Conclusion

This study reveals a tumour suppressor role of TET2 in LUAD. TET2 overexpression in A549 and H1975 cells decreased their proliferation and metastasis. In contrast, TET2 knockdown dramatically enhanced the proliferation, migration and invasion of A549 and H1975 cells. Mechanistically, activation of the cGAS-STING signalling pathway is critical for TET2-mediated suppression of the tumorigenesis and metastasis of lung cancer cells. The results of this study provide new potential molecular therapeutic targets and clinical therapies for patients with LUAD.

### Electronic supplementary material

Below is the link to the electronic supplementary material.

Supplementary Material 1

## Data Availability

The datasets used and/or analyzed during the current study are available from the corresponding author on reasonable request.
