# Supplementary material for: TET2 inhibits the proliferation and metastasis of lung adenocarcinoma cells via activation of the cGAS-STING signalling pathway
Source: BMC Cancer. 2023 Sep 4;23:825. doi: 10.1186/s12885-023-11343-x (PMC10478367; doi:10.1186/s12885-023-11343-x)

Figure 1C Dot Blot

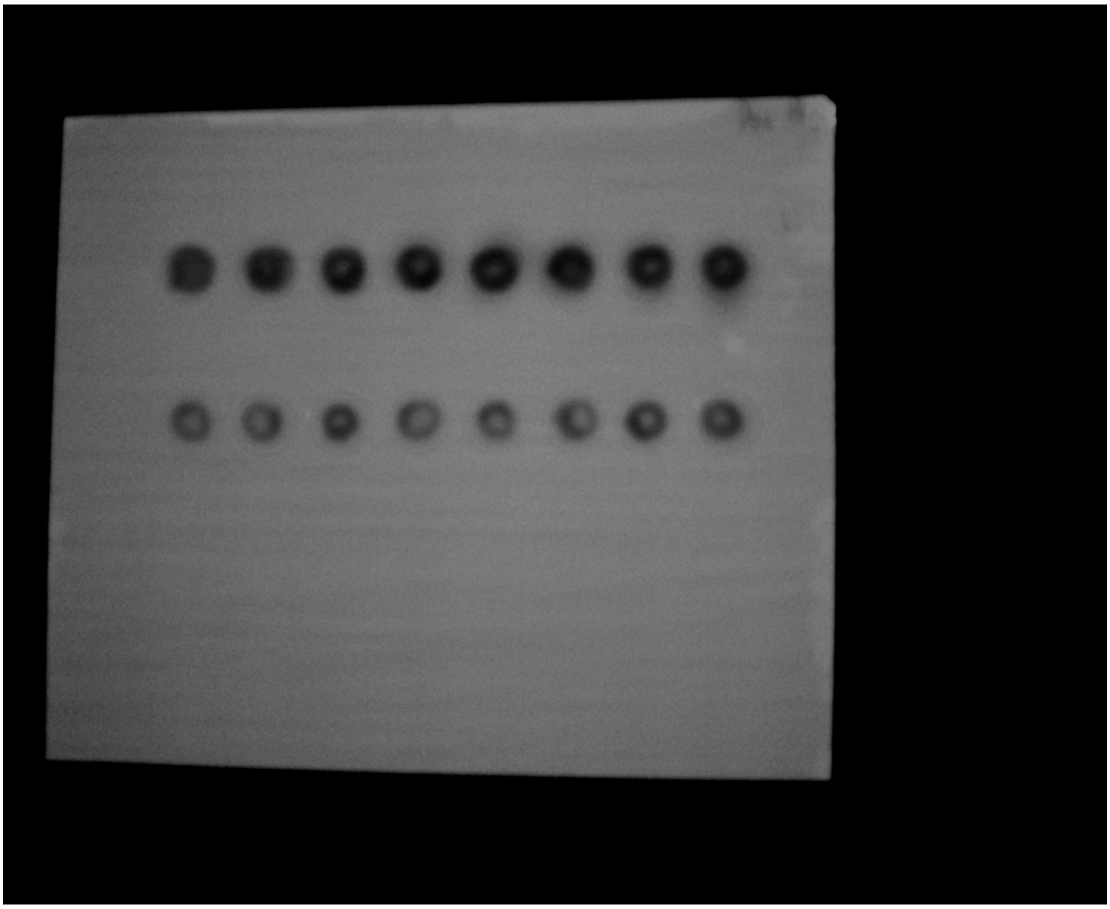

Figure 2B Western Blot-TET2 (3 repeats)

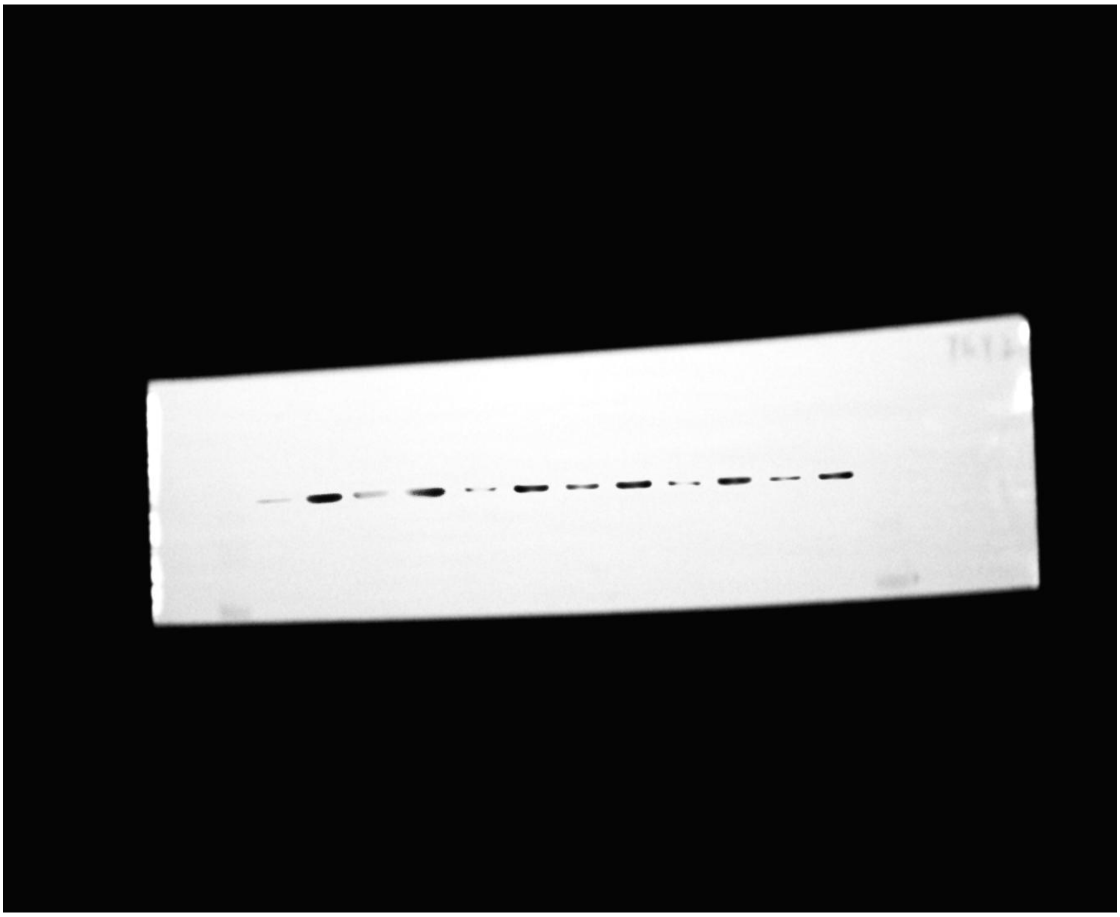

Figure 2B Western Blot-GAPDH (3 repeats)

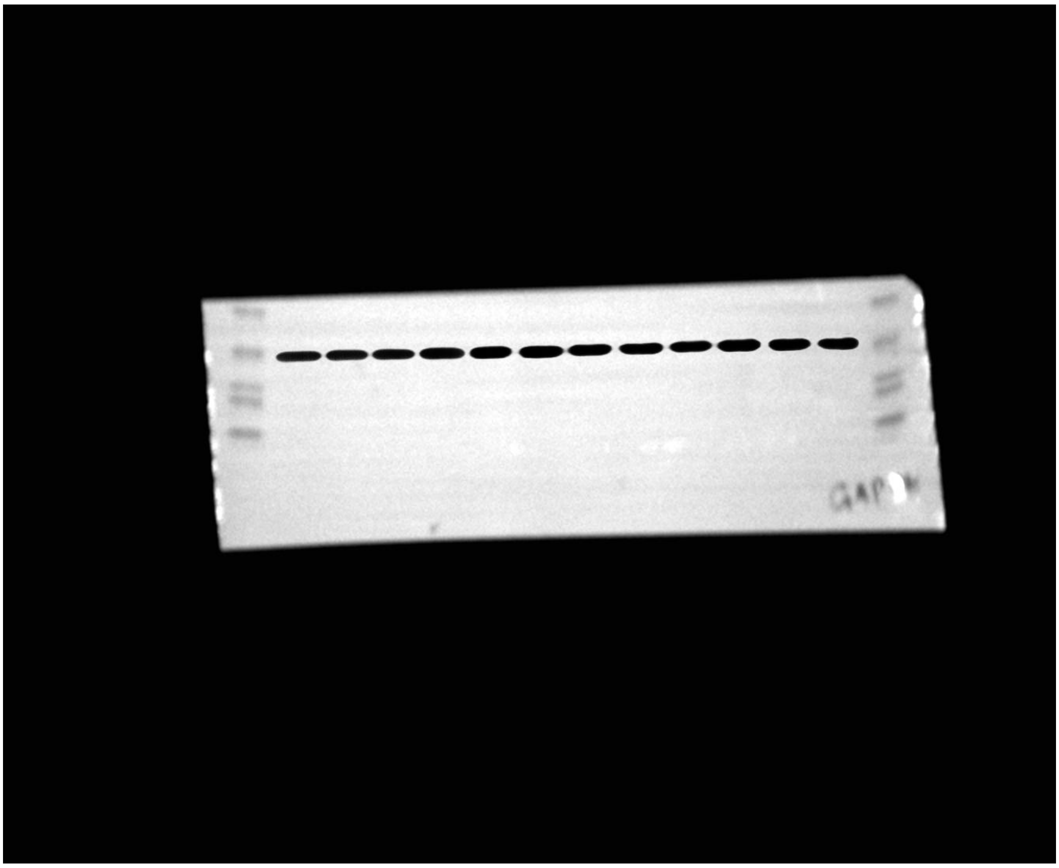

Figure 3B Western Blot-TET2 (3 repeats)

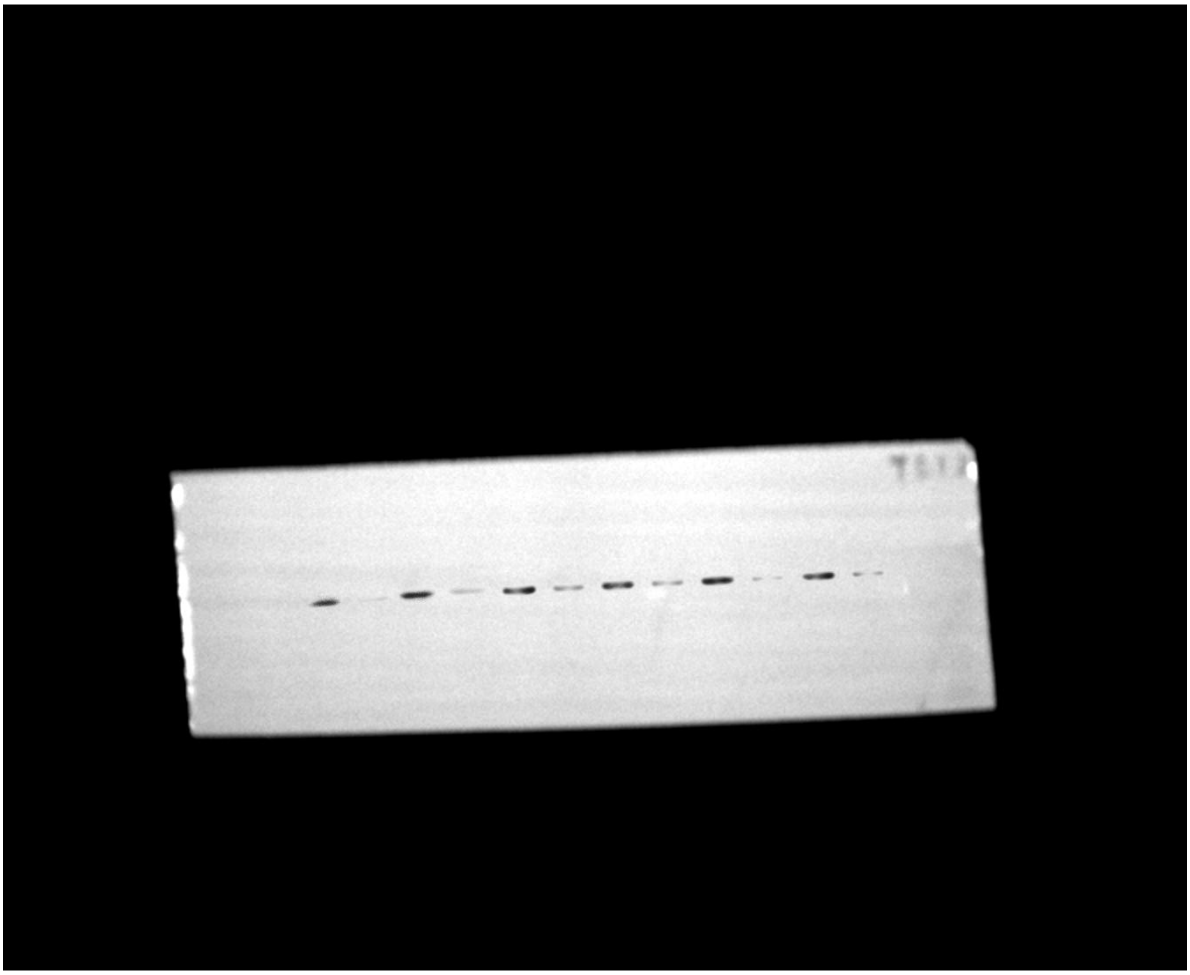

Figure 3B Western Blot-GAPDH(3 repeats)

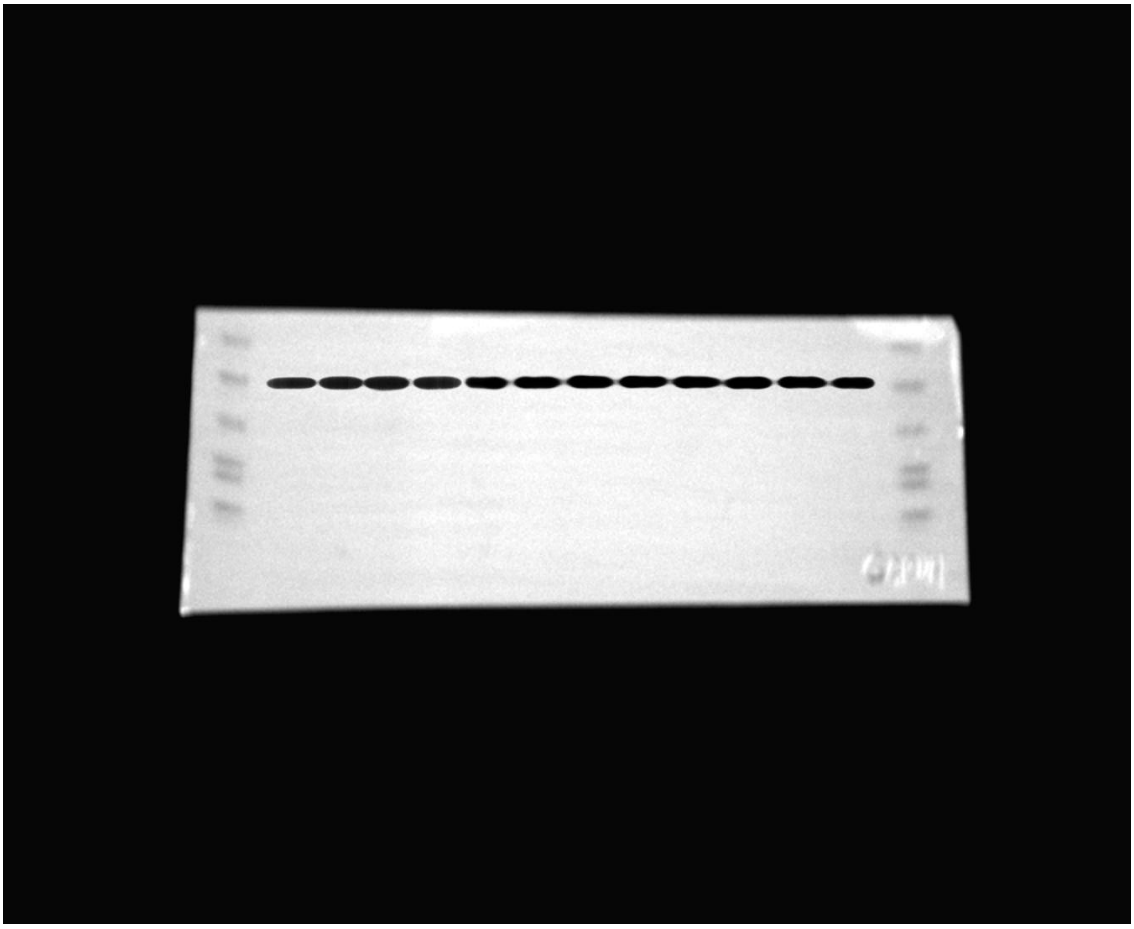

Figure 4C Western Blot-cGAS (3 repeats)

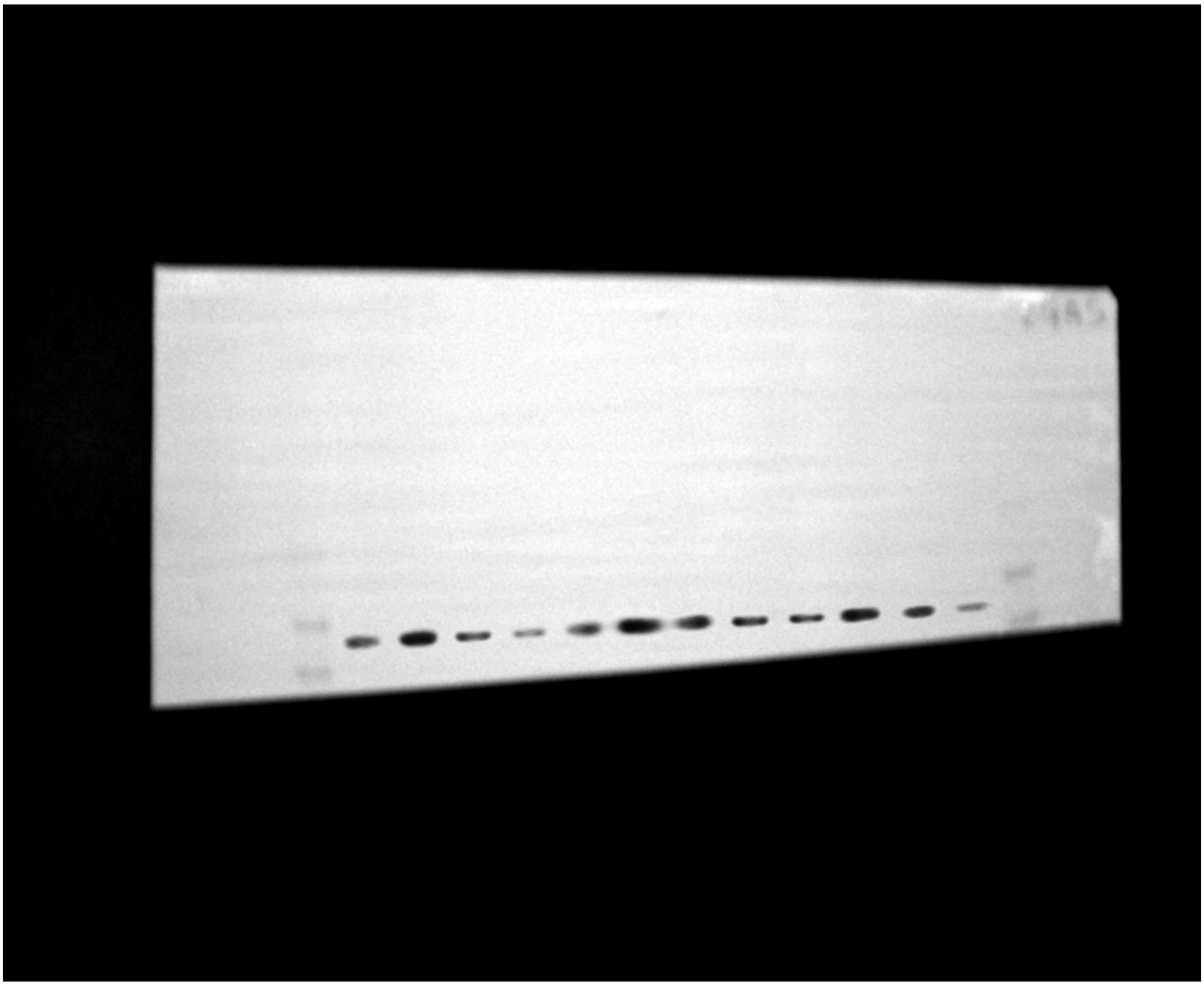

Figure 4C Western Blot-STING (3 repeats)

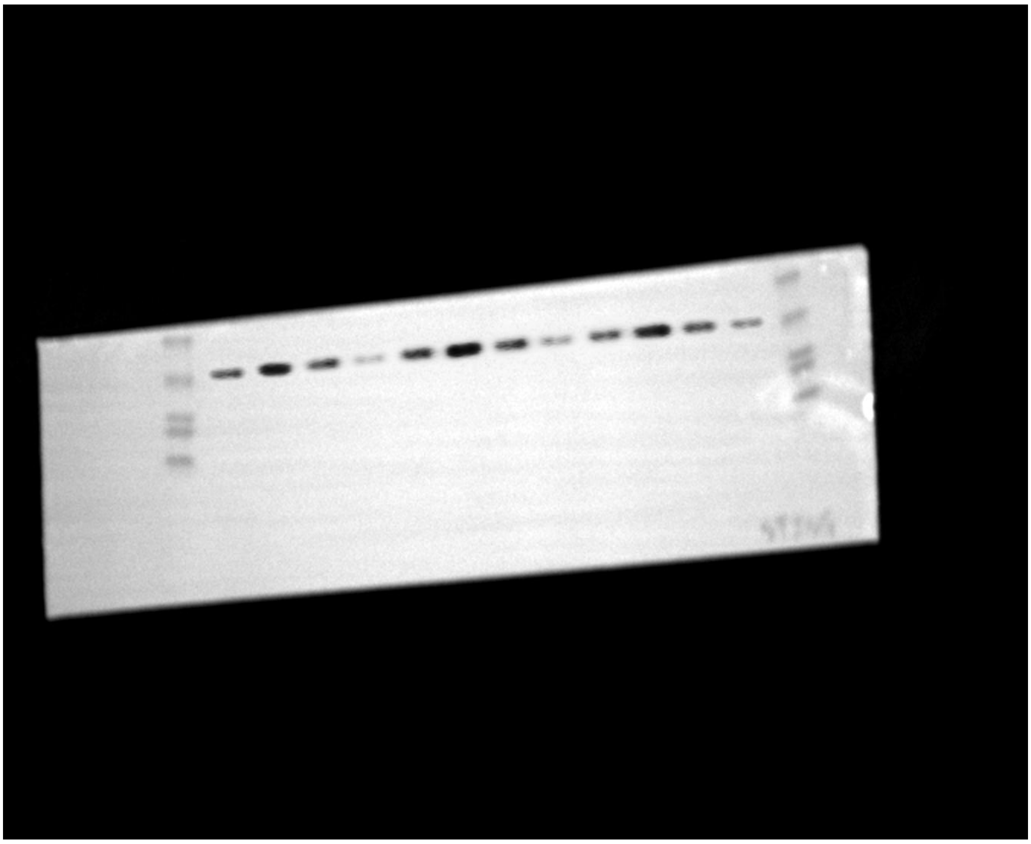

Figure 4C Western Blot-TBK1 (3 repeats)

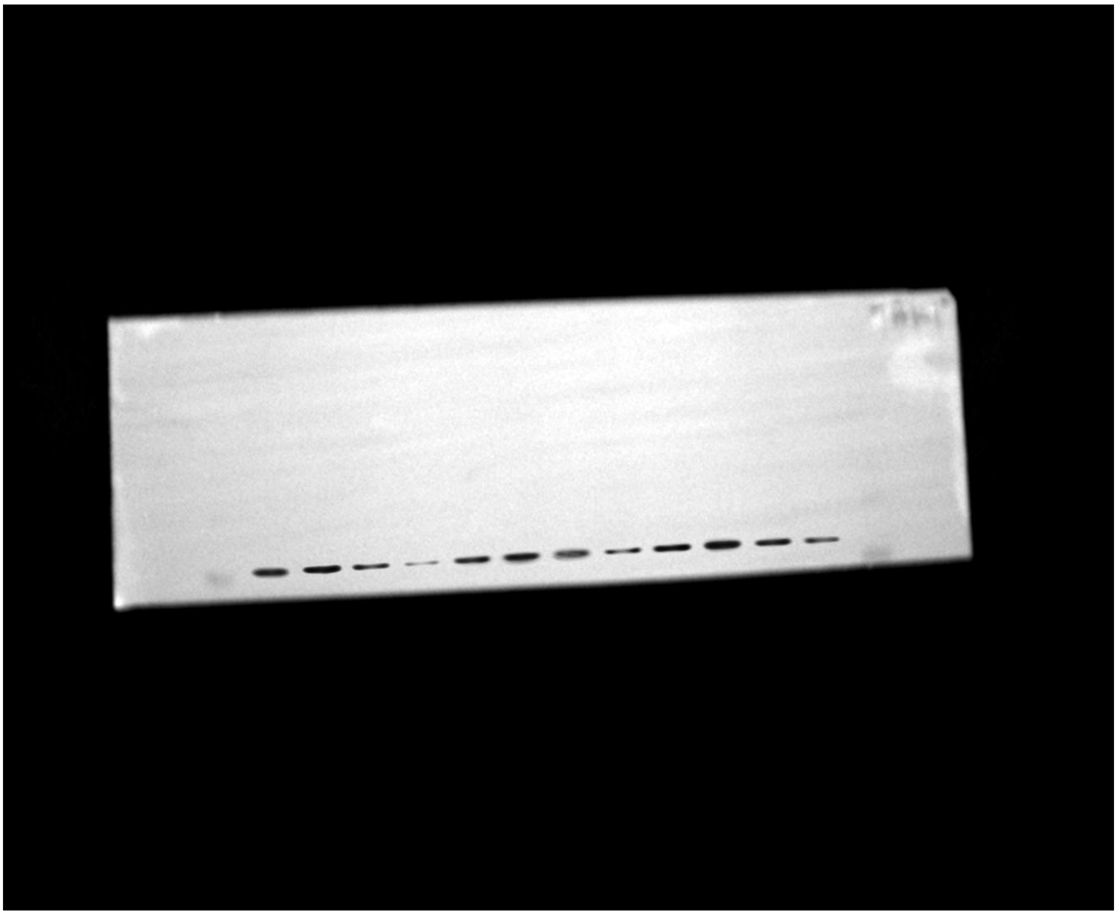

Figure 4C Western Blot-GAPDH (3 repeats)

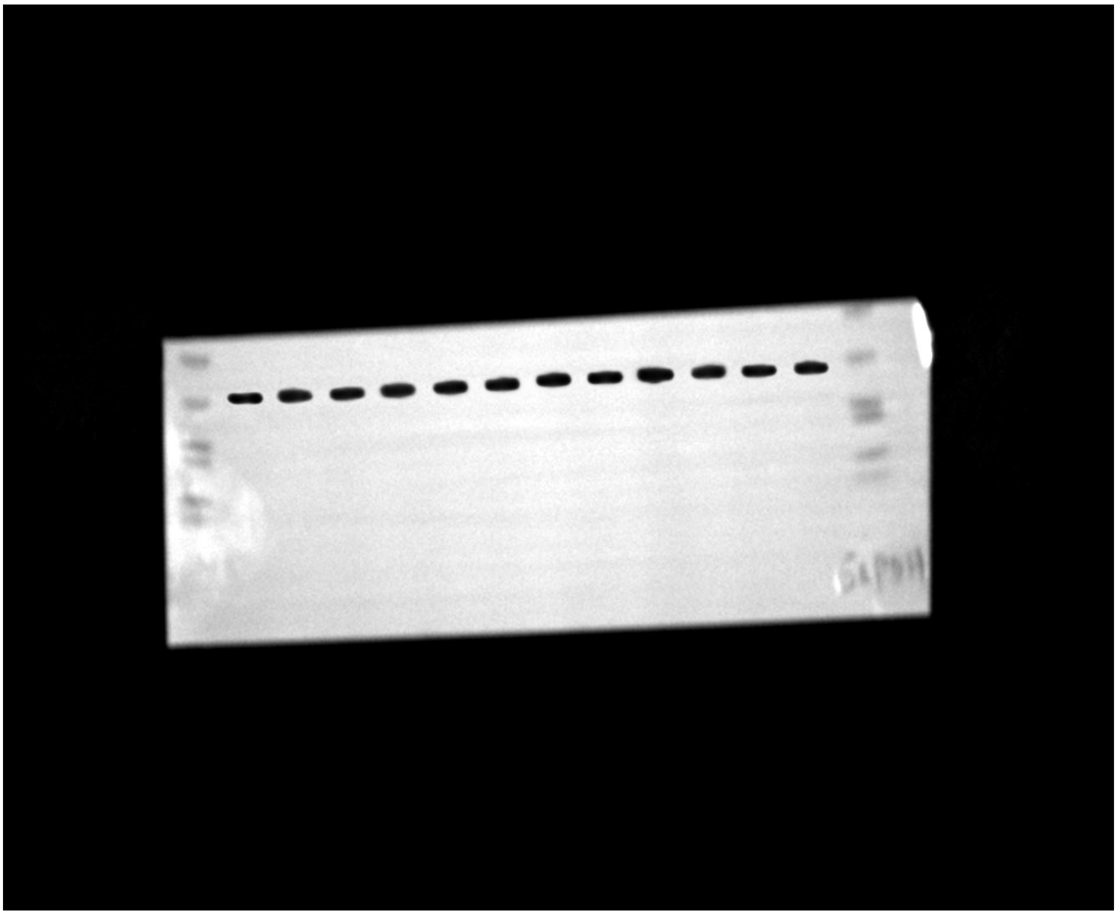

Supplement: Supplementary file 1 — Supplementary Material 1 [file 12885_2023_11343_MOESM1_ESM.pdf]
